# The complete chloroplast genome of *Iris speculatrix* Hance, a rare and endangered plant native to Hong Kong

**DOI:** 10.1080/23802359.2022.2073842

**Published:** 2022-05-13

**Authors:** Tin-Yan Siu, Kwan-Ho Wong, Bobby Lim-Ho Kong, Hoi-Yan Wu, Grace Wing-Chiu But, Pang‑Chui Shaw, David Tai-Wai Lau

**Affiliations:** a Shiu-Ying Hu Herbarium, School of Life Sciences, The Chinese University of Hong Kong; b Li Dak Sum Yip Yio Chin R & D Centre for Chinese Medicine, The Chinese University of Hong Kong; c School of Life Sciences, The Chinese University of Hong Kong; d State Key Laboratory of Research on Bioactivities and Clinical Applications of Medicinal Plants (CUHK) and Institute of Chinese Medicine, The Chinese University of Hong Kong

**Keywords:** *Iris speculatrix*, Iridaceae, rare and endangered plant, phylogenetic analysis, chloroplast genome

## Abstract

*Iris speculatrix* is a rare and endangered plant first discovered in and native to Hong Kong. The whole chloroplast genome of *I. speculatrix* is 152,368 bp in length. It contained a large single copy region (82,003 bp), a small single copy region (17,941 bp), and two inverted repeats (26,212 bp). Phylogenetic analysis of 17 species of Iridaceae was conducted. *I. speculatrix* was found to be sister to a group of 12 *Iris* species, including *I. setosa, I. lacteal*, and *I. uniflora.* The sequenced chloroplast whole genome would be useful to understand the phylogeny and to conservation of *I. speculatrix.*

## Content

*Iris speculatrix* Hance (Iridaceae) (Hance 1875), a herb with attractive purplish flower, was first discovered and published in Hong Kong and is a rare and precious plant of Hong Kong scheduled under the Forestry Regulations (Hu et al. 2003). Members of the genus *Iris* are well known for their attractive flowers and are widely applied in horticulture (Kang et al. 2020).

The sample of *I. speculatrix* was collected in Tai Tam, Hong Kong, in collaboration with Agriculture, Fisheries and Conservation Department (AFCD), The Government of the Hong Kong Special Administrative Region (HKSAR) as part of the Rare and Endangered Species project. The species is scheduled under protective legislation (and exact locality details are restricted). Hence, it is required by AFCD, The Government of the Hong Kong Special Administrative Region that the GPS should be kept confidential. The voucher specimens with collector number Rare and endangered plants (SW Shek, KW Lam, DTW Lau, and JYY Lau) 009 were deposited in the Hong Kong Herbarium, AFCD, HKSAR (https://www.herbarium.gov.hk/en/home/index.html, Dr. LAU Yuen Yung, Jenny, herbarium@afcd.gov.hk) and the Shiu-Ying Hu Herbarium, School of Life Sciences, The Chinese University of Hong Kong (https://syhuherbarium.sls.cuhk.edu.hk/, David Tai Wai Lau, syhuherbarium.sls@cuhk.edu.hk).

Fresh leaves of *Iris speculatrix* (60 mg) was used for the extraction of total genomic DNA. The sample was extracted using i-genomic Plant DNA Extraction Mini Kit (iNtRON Biotechnology, Seongnam, Gyeonggi, Republic of Korea) The manufacturer’s protocol was used. The concentration of the extract was checked using NanoDrop Lite (Thermo Fisher Scientific, Massachusetts, USA). Then, 1% agarose gel electrophoresis was used to visualize and check DNA quality. The sample was sequenced using NovaSeq 6000 platform (Illumina Inc., San Diego, CA, USA) by Novogene Bioinformatics Technology Co., Ltd. (https://en.novogene.com/, Beijing, China). A 150 bp paired-end (PE) library was constructed by Illumina sequencing. CLC Assembly Cell package v5.1.1 (CLC Inc., Denmark) was used to trim poor-quality reads (Phred score < 33).

CLC de novo assembler in CLC Assembly Cell package and SOAPdenovo v3.23 were used to assemble reads into contigs under default parameters. Gapcloser module in SOAP package was used to close gaps. After that, the contigs were aligned to the reference genome *Iris rossii* (NC_056180.1), and assembled into complete chloroplast genome. Gene annotation was obtained from GeSeq platform with reference to complete chloroplast genomes of *Iris domestica* (NC_050833.1) and *Iris gatesii* (NC_024936.1). A few adjustments were made manually for protein-coding genes, start and stop codons. The annotated genome was deposited in GenBank with the accession number OK274247.

The complete chloroplast genome of *I. speculatrix* was 152,368 bp in length, containing a large single copy (LSC) region (82,003 bp), a small single copy region (17,941 bp), and two inverted repeats (26,212 bp). The GC content was 38.04%. The chloroplast genome contained 112 unique genes, including 79 protein-coding genes, 29 tRNA genes, and 4 rRNA genes.

**Figure 1.**
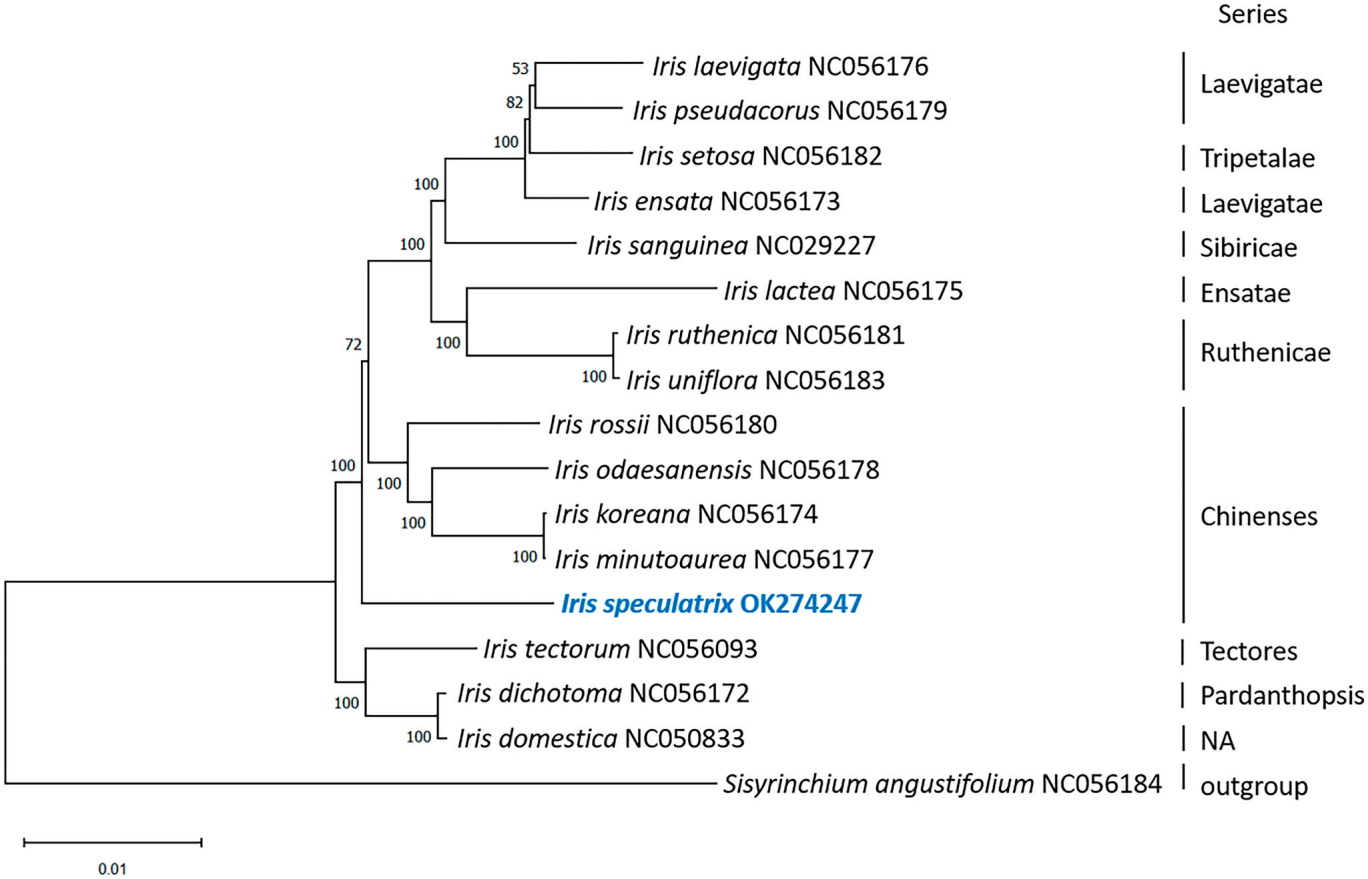
Phylogenetic tree reconstructed by maximum-likelihood (ML) analysis of 17 species.

To investigate the taxonomic position of *I. speculatrix,* the chloroplast genome was aligned with 15 other species in the genus *Iris*, and the *Sisyrinchium angustifolium* (NC056184) within Iridaceae was used as an outgroup. The complete chloroplast genomes were aligned using MAFFT 7.48 (Katoh et al. 2019). A maximum likelihood (ML) tree was constructed using MEGA X (Kumar et al. 2018) based on the best fit model GTR + G and 1000 bootstrap replicates. The phylogenetic tree was labeled with species and series (Figure 1). *I. speculatrix* was found to be sister to a group of 12 *Iris species*, including *I. setosa, I. lactea* and *I. uniflora.* In previous study of *Iris* phylogeny based on *trn*L-*trn*F and *rps*4 data, *I. speculatrix* was sister to a group of 17 *Iris* species including *I. xiphium* and *I. latifolia,* while the group was sister to another group of *Iris* containing *I. setosa, I. lactea* and *I. uniflora* (Tillie et al. 2000). From Tillie’s study, *I. speculatrix* was more distantly related to the Chinenses group of *Iris* including *I. rossii.* However, from our phylogenetic analysis, *I. speculatrix* was sister to a larger group of *Iris*, which included *I. rossii* and *I. koreana* from series Chinenses, *I. setosa* from the series Tripetalae. The species was more distantly related to *I. tectorum* from series Tectores and *I. dichotoma* from Pardanthopsis.

It was proposed that *Iris* from the series Chinenses should be classified to the new genus *Zhaoanthus* nom. provis. (Mavrodiev et al. 2014). Although it was suggested that *I. speculatrix* should be moved to *Zhaoanthus* based on morphological characters (Crespo et al. 2015; Wilson 2020), the treatment was not accepted by The World Checklist of Vascular Plants (Govaerts et al. 2021), where *Zhaoanthus speculatrix* was treated as a synonym of *I. speculatrix*.

In another study, *I. speculatrix* was also found to be outside of the Chinenses group (Guo and Wilson 2013). Tillie had suggested that *I. speculatrix* was highly sequence-divergent compared to other members of the group including *I. xiphium* and *I. latifolia*. Previous study had also suggested that *I. speculatrix* was sister to a group that included series Spuriae and Tenuifoliae (Hall et al. 2000). *I. speculatrix* was also found to be outside of series Chinenses from our study. Further study is required to resolve the taxonomic placement of *I. speculatrix* between different series among the genus *Iris*.

## Data Availability

The data that support the findings of this study are openly available in GenBank (https://www.ncbi.nlm.nih.gov) with the accession number OK274247 (https://www.ncbi.nlm.nih.gov/nuccore/OK274247.1). The associated BioProject, SRA, and Bio-Sample numbers are PRJNA796487, SRR17635354, and SAMN24866026, respectively.
